# Development of a novel analysis method for evaluating PTSD-like behavior in mice based on DSM-V criteria

**DOI:** 10.1080/10253890.2025.2612332

**Published:** 2026-01-10

**Authors:** Heather Holman, Kaylee Eggert, Ying Xiong, Paul J. Nietert, Sara J. Sidles, Ryan R. Kelly, Amanda C. LaRue, Patrick J. Mulholland, Jennifer A. Rinker, Jeffrey A. Jones

**Affiliations:** a Department of Surgery, Medical University of South Carolina, Charleston, SC, United States; b Department of Public Health Sciences, Medical University of South Carolina, Charleston, SC, United States; c Department of Pathology and Laboratory Medicine, Medical University of South Carolina, Charleston, SC, United States; d Ralph H. Johnson VA Health Care System, Research Service, Charleston, SC, United States; e Department of Neuroscience, Medical University of South Carolina, Charleston, SC, United States

**Keywords:** Posttraumatic stress disorder, analysis method, DSM-V, mouse behavior, modified single prolonged stress, inescapable foot shock

## Abstract

Posttraumatic stress disorder (PTSD) occurs after exposure to a traumatic event, leading to debilitating symptoms, including avoidance, hypervigilance, and functional impairment. There is a paucity of effective therapies to treat PTSD, partially due to the difficulty in identifying consistent underlying mechanisms. Using a modified single prolonged stress (mSPS) paradigm combined with single housing to induce both acute fear conditioning and chronic stress in mice, we developed a novel analysis method to robustly define a PTSD-like phenotype based on the criteria from the Diagnostic and Statistical Manual of Mental Disorders, 5th Edition (DSM-V). Following mSPS exposure, C57BL/6NHsd mice underwent behavioral testing to examine each of the criteria of PTSD according to the DSM-V. Specific parameters with the largest effect sizes between mSPS and non-mSPS mice were chosen. Absolute *z*-scores were generated for each behavioral parameter, and mSPS mice whose *z*-scores were outside the 85th confidence interval for at least one parameter for each of the eight criteria were defined as susceptible; the remainder of the exposed mice were considered resilient. Finally, resilient mice were evaluated for anhedonia and hyperlocomotive behaviors. The results demonstrated that a PTSD-like phenotype can be robustly defined in mice based on all 8 DSM-V criteria. Importantly, 29.76% of mSPS mice were classified as susceptible, which is similar to the incidence observed in humans exposed to trauma. This novel behavioral analysis method may assist in better defining a PTSD-like phenotype, identifying a more robust population, which may help facilitate the discovery of the underlying mechanism(s) of PTSD and its association with other comorbidities.

## Introduction

Posttraumatic stress disorder (PTSD) is a psychological disorder that occurs after a direct, witnessed, or repeated exposure to aversive details of a traumatic experience. The lifetime prevalence of PTSD in the civilian population is approximately 4%–7%; however, it can reach as high as 17% in the veteran population and 36% in sexual assault survivors. The incidence of PTSD can vary based on the type of trauma, but on average, about one in four individuals who are exposed to a traumatic experience develop PTSD (Breslau et al., 1991; Schein et al., 2021). PTSD can be chronic and debilitating, consisting of avoidance symptoms, negative alterations in mood and cognition, hypervigilance, and functional impairment (Center for Substance Abuse Treatment, 2014). It is diagnosed by clinicians based on the following criteria outlined in the Diagnostic and Statistical Manual of Mental Disorders, 5th Edition (DSM-V): A) exposure to a threatening stimulus; B) presence of intrusion symptoms; C) continued avoidance of stimuli; D) negative alterations in mood and cognition; E) hyperarousal and reactivity; F) symptoms of all criteria lasting more than one month posttraumatic experience; G) impairment of social, occupational, and other important life activities; and H) symptoms that cannot be attributed to substances or other medical conditions (Center for Substance Abuse Treatment (US), 2014). Patients must meet all eight criteria to obtain a diagnosis of PTSD. Patients who do not meet all eight criteria may obtain other psychiatric diagnoses following the traumatic experience, such as depression and generalized anxiety (Ayazi et al., 2014; Wang et al., 2023). Treatment for PTSD consists of both cognitive behavioral therapy as well as pharmacological therapy, most commonly with selective serotonin reuptake inhibitors (SSRIs). However, the prognosis is quite poor, where only 38% of patients recover from PTSD, and of those that do recover, 30% ultimately experience a reoccurrence of PTSD symptoms (Committee on the Assessment of Ongoing Efforts in the Treatment of Posttraumatic Stress Disorder, 2014; Merians et al., 2023). Importantly, individuals with a PTSD diagnosis are at increased risk of developing other psychiatric diseases, such as substance and alcohol misuse and major depressive disorder, as well as physiological conditions, such as cardiovascular disease and autoimmune diseases (Edmondson & von Kanel, 2017; O’Donovan et al., 2015; Ryder et al., 2018; Sareen, 2014). The lack of appropriate and successful treatment options primarily stems from a poor understanding of the underlying mechanisms of PTSD, and few studies have addressed how PTSD can exacerbate other comorbidities. This is due, in part, to the variety of analysis methods and the difficulty in defining PTSD in preclinical and translational rodent models, which is supported by the systematic review conducted by Ferland-Beckham et al. (Ferland-Beckham et al., 2021; O’Hara et al., 2025; Richter-Levin et al., 2019; Verbitsky et al., 2020).

Currently, PTSD-like phenotypes in rodents are defined and analyzed by a variety of methods. Some groups focus more on the fear learning aspects of PTSD, while others focus on reactions to uncontrollable stress (Deslauriers et al., 2018; Wang et al., 2008; Wendelmuth et al., 2020; Zhang et al., 2021). Additionally, very few research groups address all 8 DSM-V categories needed to define PTSD, which in rodents requires both directed experimental design and behavioral analysis (Perrine et al., 2016; Wang et al., 2022; Xi et al., 2023; Xiao et al., 2024). For example, traumatization can be induced in a single day or across multiple days through a variety of methods, including social, psychological, or physical stressors (Borghans & Homberg, 2015; Deslauriers et al., 2018; Sidles et al., 2024; Verbitsky et al., 2020). Additionally, few groups have studied persistent rodent responses to stress induction that occur after 4-weeks (Deslauriers et al., 2018; Perrine et al., 2016; Wang et al., 2008; Wang et al., 2022; Wendelmuth et al., 2020; Xi et al., 2023; Xiao et al., 2024; Zhang et al., 2021). Many groups similar to Xi et al., have examined the PTSD-like phenotype 7 days following a modified single prolonged stress (mSPS) induction, whereas others, such as Wang et al. and Zhang et al., have assessed PTSD-like behaviors 14 days following mSPS induction (Wang et al., 2022; Xi et al., 2023; Zhang et al., 2021). Additionally, there is little agreement on what characteristics should be tested and what behavioral measures should be collected. For instance, Perrine et al. and Xiao et al. primarily utilized fear extinction/conditioning as a readout for PTSD-like behavior, whereas Wang and colleagues incorporated the open field and elevated plus maze along with fear conditioning/extinction as a measure of anxiety-like behavior in PTSD-like mice (Perrine et al., 2016; Wang et al., 2022; Xiao et al., 2024). Torrisi et al. developed an arousal-based individual screening tool using z-normalization to define susceptible and resilient mice following traumatization based on a single behavioral test, acoustic startle reactivity; whereas Colucci et al. utilized the open field arena and social interaction test to separate susceptible versus resilient animals by conducting a correlation analysis between the two tests (Colucci et al., 2020; Torrisi et al., 2021). While this in no way invalidates these previous studies or the data they have collected, it highlights the lack of consensus on how to accurately and consistently induce a PTSD-like phenotype in rodents and, importantly, how to analyze the multiple behavioral deficits to define a PTSD-like phenotype.

In the present study, we utilized a modified SPS model (mSPS) to induce a PTSD-like phenotype based on the repeated foot shock paradigm described by Sidles et al., combined with a single prolonged stress (SPS) model (Liberzon et al., 1997; Serova et al., 2013; Sidles et al., 2024). This was designed to incorporate both the associative fear learning and hyperarousal induced by repeated foot shock and the prolonged, uncontrollable stressors of traumatic exposure introduced by SPS. By combining these models, we hoped to capture both the acute fear-conditioning mechanisms and the chronic stress-induced dysregulation of the hypothalamic–pituitary–adrenal (HPA) axis, thereby producing a more salient and translationally relevant PTSD-like phenotype. Importantly, we have described a novel analytical method to define a susceptible phenotype robustly in mice that incorporates both experimental design and behavioral analysis and utilizes all eight criteria identified in the DSM-V.

## Methods

### Animals

All procedures were approved by the Ralph H. Johnson VA Health Care System Institutional Animal Care and Use Committee and followed the Guide for the Care and Use of Laboratory Animals (National Research Council (US) Committee for the Update of the Guide for the Care and Use of Laboratory Animals, 2011). Wild-type male and female C57BL/6NHsd mice (*n* = 122, Inotiv, catalog #: 044-US) at 10–14 weeks of age were utilized. The control mice were housed in groups (2–5 animals per cage), and the mSPS mice were single housed following the induction paradigm. The animals had access to food and water *ad libitum*. Husbandry rooms were set to a 12-h light cycle (6:00 am lights on, 6:00 pm lights off) and were kept at approximately 20 °C and 50% humidity. Cages were changed once per week by the same researcher throughout the study.

### Modified single prolonged stress model

The modified single prolonged stress (mSPS) paradigm consists of repeated foot shock immediately followed by single prolonged stress events (2-h restraint, 20-min group swimming, and loss of consciousness (1–2 min induced by isoflurane)) and single housing. This protocol was adapted from the SPS protocol described by Liberzon et al. combined with the repeated foot shock protocol described by Sidles et al. (Liberzon et al., 1997; Sidles et al., 2024). The mice (*n* = 84) were singly placed into fear conditioning chambers (Noldus Leesburg, VA) and exposed to 5-foot shocks (1.0 mA, 1-s duration) 1 min apart that was preceded by a 20-s tone (80 dB, 9 kHz) for a total of 6 min. Following repeated foot shock, the mice were placed individually into a 50 mL conical tube with air holes placed in both the cap and sides of the tube to restrict movement for 2 h. Following the restraint procedure, the mice underwent a group swimming event of 3–8 mice for 20 min at room temperature with a water depth of approximately 25 cm. Following the group swimming event, the mice were dried with a towel and heat lamp, placed in the chambers, and exposed to isoflurane until loss of consciousness (1–2 min). At the end of the mSPS, the mice were single-housed and left undisturbed for 7 days. The mice remained single-housed for the rest of the experiment. Control mice (*n* = 38) were not exposed to the mSPS paradigm and remained group-housed throughout the study.

### Behavioral assessments

**Figure 1.**
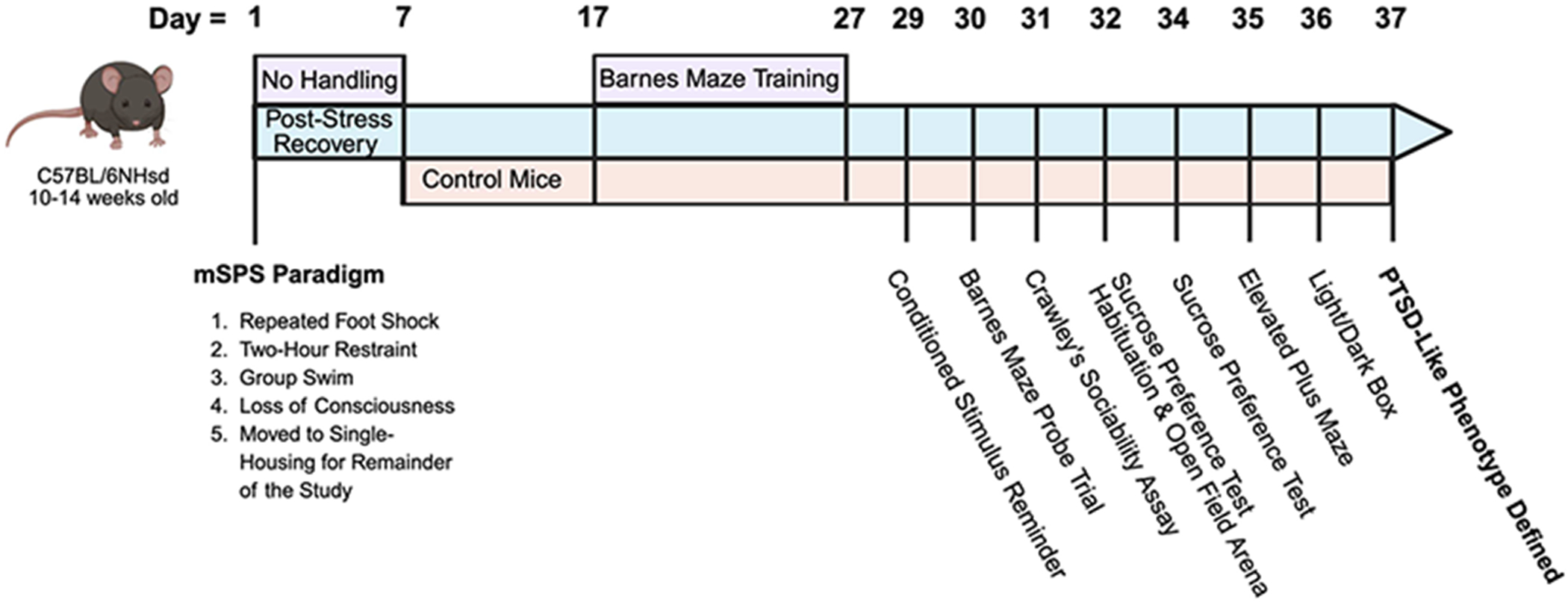
Traumatization and behavioral testing timeline. PTSD was induced in C57BL/6NHsd mice through a modified single-prolonged stress (mSPS) paradigm consisting of repeated foot shock (5 × 1-s foot shocks at 1.0 mA over 6 min, each preceded by 20 s, 80 dB, 9 kHz tone (conditioned stimulus)) followed by 2-h restraint, 20 min group swimming, and loss of consciousness induced by isoflurane (1–2 min). The mice were single housed for the remainder of the study following the mSPS paradigm. Four weeks post-mSPS paradigm, the mice underwent a series of behavioral tests lasting 17 days to assess the PTSD-like phenotype. The control mice did not undergo the mSPS paradigm and remained group-housed for the entire study but underwent the same handling and behavioral testing starting on day 17 as the mSPS mice. Figure created with BioRender.com.

Mice underwent 8 days of behavioral testing starting 29 days post-mSPS paradigm. The control mice were subjected to behavioral testing at 14–18 weeks of age, and underwent the same behavioral testing as the mSPS mice. The order of behavioral testing remained the same throughout the studies and consisted of conditioned stimulus reminder test, Barnes maze probe trial, Crawley’s sociability test, open field arena test, sucrose preference test, elevated plus maze test, and light/dark box test (Figure 1). Only one behavioral assay was conducted per day, with the exception of the sucrose preference test, in which habitation began on the same day that the mice participated in the open field arena. Training for the Barnes maze started 17 days post-mSPS induction paradigm and finished prior to beginning behavioral testing. Noldus EthoVision XT was utilized to collect video footage of the mice during behavioral testing and for analysis of mouse behavior. All behavioral assessments were conducted during daylight hours (6:00 am-6:00 pm) under ambient light unless otherwise specified. All the mice were habituated to the room for 30 min prior to behavioral testing.

#### Conditioned stimulus reminder test

The mice were individually placed into the fear conditioning chambers (chamber: 50 cm (width) × 40 cm (depth) × 50 cm (height), cage: 17 cm (width) × 17 cm (depth) × 25 cm (height), Noldus, Leesburg, VA) for 6 min and were exposed to the conditioned stimulus/tone (80 dB, 9 kHz, 20-s duration) after 3 min without foot shock. The following parameters were analyzed: distance moved (cm), time spent inactive (s or %), and mean velocity (cm/s) over the course of the entire 6 min, as well as before, during, and after the conditioned stimulus/tone.

#### Barnes maze

The Barnes Maze apparatus (92 cm apparatus diameter, 5 cm hole diameter, Noldus, Leesburg, VA) was placed under bright light (~690 lux), with three visual cues (red square, yellow triangle, and blue circle) placed around the walls of the maze. The subject mouse was allowed to habituate to the maze by first being placed in the escape tunnel for 1 min and then being placed in the center of the maze for 5 min or until the mouse found the escape tunnel. The mouse was returned to its home cage for 1 h, and the acquisition phase started. The escape tunnel was moved to a different location from the habituation period and remained in that position for the duration of the acquisition phase and probe trial. The acquisition phase lasted 10 days, with two trials per day in which the mouse was placed in the center of the maze and allowed to freely explore the maze for 3 min or until it found the escape tunnel. The mouse was placed in its home cage for at least 1 h before starting the second acquisition trial for the day. The probe trial occurred three days after the end of the acquisition phase. During the probe trial, the escape tunnel was removed. Each mouse was placed in the center of the maze and had up to 1 min to find the previous location of the escape tunnel. The following behavioral parameters were collected: duration until the mouse found the entry zone of the escape tunnel (s), distance traveled until the mouse found the entry zone of the escape trial (cm), number of incorrect holes the mouse checked, total number of errors the mouse made, including repeat visits to incorrect holes, discovery of the escape tunnel within the 1 min period, and mean velocity (cm/s) (Pitts, 2018).

#### Crawley’s sociability test

Mice were placed in the center compartment of a 3-chamber arena [60 cm (width) × 40.5 cm (depth) × 22 cm (height)], with empty wire cups [10 cm (diameter) × 20 cm (height) Noldus, Leesburg, VA] and allowed to habituate for 5 min. An unfamiliar sex-matched adult mouse was placed in the wire cup in the right compartment, and the wire cup in the left compartment remained empty. The mice were allowed to explore the entire arena for 10 min freely. Then, another unfamiliar sex-matched adult mouse was placed in the wire cup in the left compartment, and the subject mouse was allowed to explore the entire arena for another 10 min freely. The following behavioral parameters were collected during the second and third trials: time spent sniffing the familiar mouse (s), time spent sniffing the novel mouse (s), time spent sniffing the empty cup (s), latency to first approach the familiar mouse (s), latency to first approach the novel mouse (s), and latency to first approach the empty cup (s) (Kaidanovich-Beilin et al., 2011).

#### Open field arena

The mice were placed in the center of the open field arena (40 cm (width) × 40 cm (depth) × 30 cm (height), Noldus, Leesburg, VA) and allowed to explore the arena for 5 min freely. The following behavioral parameters were collected: average velocity (cm/sec), total distance traveled (cm), duration in the center of the arena (s), and duration at the arena’s edge (s) (Seibenhener and Wooten, 2015). The open field arena was not utilized to define the susceptible phenotype in mSPS mice but was used to assess hyperlocomotion in resilient mice.

#### Sucrose preference test

Mice were kept in home cages and habituated to two water bottles filled with 250 mL of reverse osmosis water for 48 h. After 48 h, the amount of water each mouse consumed was recorded, and one bottle was replaced with 250 mL of a 2% (w/v) sucrose solution in reverse osmosis water. After 24 h, the amount of reverse osmosis water (mL) and 2% sucrose solution each mouse consumed was recorded (mL) (Liu et al., 2018). The body weights of each mouse were recorded at the start of the test and after 24, 48, and 72 h. The bottles were frequently checked throughout the test to ensure that no leaks occurred.

#### Elevated plus maze

The mice were placed in the center of the elevated plus maze (75 cm (arm-to-arm length) × 5 cm (arm width) × 40 cm (closed arm height), 101 cm (height above ground) Noldus, Leesburg, VA) facing the closed arms at the start of the trial. The mice were allowed to explore the maze for 5 min freely. Any mouse that fell off the maze was returned to the center of the maze facing the closed arms. The following behavioral parameters were quantified: duration in the closed arms (s), open arms (s), and center of the maze (s) (Walf & Frye, 2007).

#### Light/dark box

The light/dark apparatus (enclosed compartment: 17.5 cm (width) × 40 cm (depth) × 35 cm (height), light compartment: 25 cm (width) × 40 cm (depth) × 35 cm (height), Noldus, Leesburg, VA) was placed on an elevated table. The mice were placed in the center of the open compartment of the box at the start of the trial and were allowed to explore the apparatus for 5 min freely. The following behavioral parameters were analyzed: duration in the light compartment of the box (s), duration in the dark (enclosed) compartment of the box (s), and frequency of entry into the enclosed compartment of the box (Costall et al., 1989).

### Identification of parameters to define susceptible phenotype

A susceptible phenotype was defined in mice through behavioral assessments 4 weeks after they underwent the mSPS paradigm. Based on work published by Verbitsky et al., behavioral tests were uniquely chosen to meet each of the human criteria for a PTSD diagnosis according to the DSM-V (Table 1) (Verbitsky et al., 2020). These criteria also address the 4 Research Domain Criteria (RDoC) constructs altered in PTSD, which include negative valence, positive valence, cognitive processes, and arousal (Table 2) (Richter-Levin et al., 2019; Schmidt & Vermetten, 2018). The behavioral data of male and female mice were analyzed separately based on potential sex-dependent differences in response to the modified mSPS paradigm (Keller et al., 2015; Mancini et al., 2021; Pooley et al., 2018), and several parameters were measured for each behavioral test. Behavioral data were assessed for normality using the D’Agostino–Pearson test, and any data not normally distributed underwent the appropriate transformation (conditioned stimulus removal test: log-transformed pretone and posttone duration inactive; Crawley’s sociability assay: log-transformed latency to approach novel mice; elevated plus maze test: square root-transformed time spent in open arms versus closed arms). Glass delta effect sizes ($\text{effect size}=\frac{\mu_{\mathrm{ST}}-\mu_{\mathrm{SC}}}{\sigma_{\mathrm{SC}}},$ where *μ*_ST_ = mean of SPS animals, *μ*_SC_ = control mean, *σ*_ST_ = control starndard deviation) were calculated for each parameter for each behavioral test in order to determine which behavioral parameters were altered most significantly between mSPS and control animals. The phi coefficient ($\phi =\sqrt{\frac{\chi^{2}}{N}},$ *χ*^2^ = chi square statistic, *N* = total sample size) was VN’ used to determine the effect size for categorical variables (Barnes Maze). At least two independent parameters with the highest effect size for each measurable criteria of PTSD from the DSM-V (criteria B, C, D, E, and G) were then chosen to define a susceptible behavioral phenotype.

### Defining a susceptible phenotype

Absolute *z*-scores for each mouse were generated for each parameter using the control mean and standard deviation to account for population variability ($z\text{-score}=\frac{\chi_{\text{T}-\mu_{\mathrm{SC}}}}{\sigma_{\mathrm{SC}}},$ where *χ* = value for individual SPS animal, *μ*_SC_ *μ*_SC_ = control mean, and *σ*_SC_ = control standard deviation). Mice with *z*-scores above the 85% confidence interval (*z*-score = 1.44) for at least one behavioral parameter for each of the five measurable criteria of PTSD according to the DSM-V (Table 1) were defined as having a susceptible behavioral phenotype and were referred to as “susceptible” mice. The mice that underwent the mSPS paradigm but were not categorized as having a susceptible phenotype since the mice had *z*-scores greater than 1.44 on 4 or fewer criteria were referred to as “resilient” mice. Absolute *z*-scores for each behavioral parameter for the control mice were calculated, and none of the control mice scored above the 85% confidence interval on all five measurable criteria of PTSD (Table 1). Therefore, none of the control mice were considered to have a susceptible phenotype.

### Evaluation of anhedonia and hyperlocomotion

Behavioral data for mice that were not classified as susceptible mice (resilient mice) were re-evaluated for anhedonia and hyperlocomotive behaviors. The sucrose preference test was utilized to determine anhedonia, and the open-field arena was used to characterize hyperlocomotion. The data were separated by sex, and *z*-scores for sucrose consumed/body weight (anhedonia) and hyperlocomotion were generated for each mouse using the control mean and control standard deviation. Mice with *z*-scores above the 85% confidence interval (*z*-score = 1.44) were considered to have aberrant sucrose consumption, hyperlocomotion, or both aberrant sucrose consumption and hyperlocomotion.

### Statistical analysis

Statistical analysis was performed using GraphPad Prism version 10.1.2 (GraphPad Software, Boston, MA). All the data were assessed for normality using the D’Agostino–Pearson test. Any data not normally distributed underwent an appropriate transformation (conditioned stimulus reminder test: log-transformed pretone and posttone duration inactive; Crawley’s sociability assay: log-transformed latency to approach a novel mouse, elevated plus maze test: square root-transformed time spent in open arms versus closed arms, open field arena: square root-transformed average velocity). All data are shown as the mean ± standard error of the mean (SEM). Two-way analysis of variance (ANOVA) with Tukey’s multiple comparisons test was performed on the behavior data to define a susceptible phenotype, including the conditioned stimulus reminder test, elevated plus maze, light/dark box, sucrose preference test (except for sucrose consumption normalized to total liquid consumed), and Crawley’s sociability assay. The Kruskal–Wallis test was performed on the sucrose consumed normalized to the total liquid consumed from the sucrose preference test, as the normalized data were nonparametric. Fisher’s exact test was performed on the Barnes maze data, as it is a categorical variable. One-way ANOVA with Tukey’s multiple comparison test was performed on the behavior data for defining anhedonic and hyperlocomotive behavior, including the open field arena and sucrose consumed normalized to total body weight. The significance threshold for all the statistical tests was set using an *α*-value of 0.05.

## Results

### Identification of behavioral parameters to define susceptible phenotype

Four weeks post-mSPS paradigm, all the mice underwent a battery of behavioral tests in order to determine the baseline behavior of the control mice and to separate resilient mice from those that possessed a susceptible phenotype (Figure 1). To determine which behavioral tests and parameters differentiated resilient and susceptible mice, the effect size for each parameter associated with each specific behavioral assessment was calculated utilizing the control mean and standard deviation. Behavioral parameters with effect sizes greater than 0.5 were selected as potential parameters for differentiating resilient versus susceptible mice (Table 1) (Verbitsky et al., 2020). An effect size greater than 0.5 was chosen as the cutoff, as effect sizes larger than 0.5 indicate a moderate to large effect (Lakens, 2013; Sullivan & Feinn, 2012). At least two independent parameters with the largest effect sizes were selected for each of the criteria.

**Figure 2.**
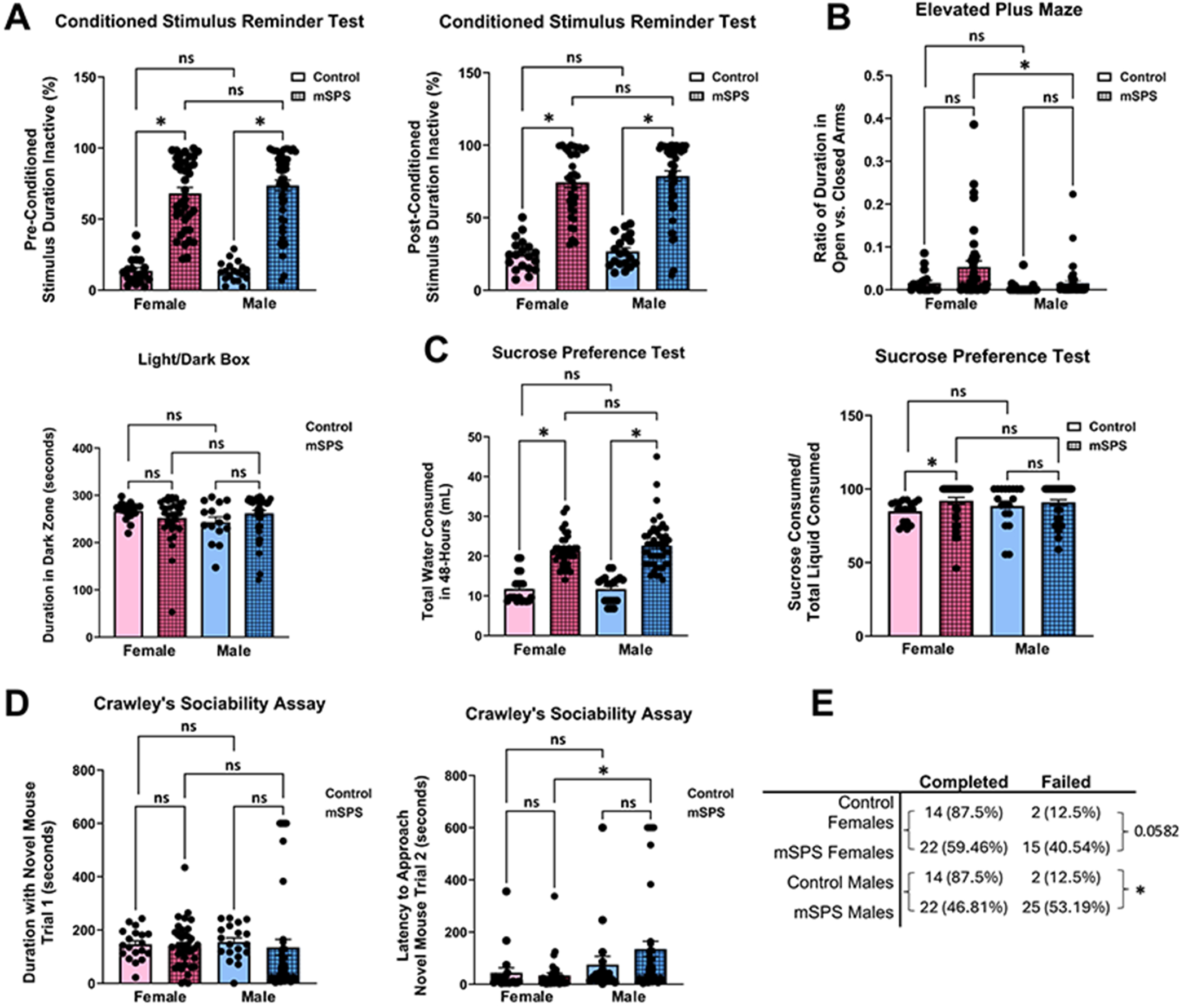
Behavioral assessments separated by sex. (A) Four weeks post-mSPS, both male and female mSPS mice displayed increased freezing behavior before (effect of trauma *p*-value < 0.0001, effect of sex *p*-value = 0.8125, effect of interaction *p*-value = 0.8609) and after the conditioned stimulus (9 kHz, 80 db, 20-s tone) (effect of trauma *p*-value < 0.0001, effect of sex *p*-value = 0.3929, effect of interaction *p*-value = 0.5939). (B) mSPS female mice also exhibited risky behavior, as evidenced by more time spent in the open versus closed arms in the elevated plus maze (effect of trauma *p*-value = 0.0182, effect of sex *p*-value = 0.0011, effect of interaction *p*-value = 0.4248). There were no differences in the duration spent in the dark zone on the light/dark box (effect of trauma *p*-value = 0.8721, effect of sex *p*-value = 0.7096, effect of interaction *p*-value = 0.1530). (C) Male and female mice 4 weeks post-mSPS had increased water consumption (effects of trauma *p*-value < 0.0001, effects of sex *p*-value = 0.4981, effects of interaction *p*-value = 0.4667), and female mSPS mice had increased sucrose consumption (*p*-value = 0.0102, Kruskal–Wallis statistic = 11.3). (D) In Crawley’s sociability assay, there was no difference in the amount of time spent with the novel mouse in trial 2 (effect of trauma *p*-value = 0.6095, effect of sex *p*-value = 0.9165, effect of interaction *p*-value = 0.7979) and took longer to approach the novel mouse in trial 3 (effect of trauma *p*-value = 0.6691, effect of sex *p*-value = 0.0013, effect of interaction *p*-value = 0.3063). (E) mSPS mice were less likely to find the previous location of the escape tunnel, thus failing the Barnes maze probe trial (data is displayed as mean ± SEM, A–D: two-way ANOVA, or one-way ANOVA, *p-value < 0.05, control females *n* = 16–19, mSPS females *n* = 37, control males *n* = 16–19, mSPS males *n* = 47; E: Fisher’s Exact test, *p-value < 0.05).

Among the parameters chosen, mSPS mice displayed an increase in freezing behavior as measured by inactivity both before and after the conditioned stimulus (9 kHz, 80 db, 20-s tone), demonstrating a lasting fear response to both the conditioned stimulus and unconditioned stimulus (foot shock chamber) (Figure 2A). Interestingly, female mSPS mice showed higher risk-taking behavior in the elevated plus maze, as evidenced by an increase in the ratio of time spent in the open versus closed arms of the elevated plus maze (Figure 2B). Furthermore, the mSPS mice consumed more water during sucrose preference test habituation, and the female mSPS mice consumed more sucrose than the control mice (Figure 2C). Male mSPS mice took longer to approach the novel mouse in Crawley’s sociability assay, indicating social impairment following the mSPS paradigm (Figure 2D). Finally, the proportion of male and female mSPS mice that were able to find the previous location of the escape tunnel on the Barnes maze was significantly lower than that of control mice, demonstrating cognitive impairment in mSPS mice (Figure 2E).

### Definition of susceptible phenotype

**Figure 3.**
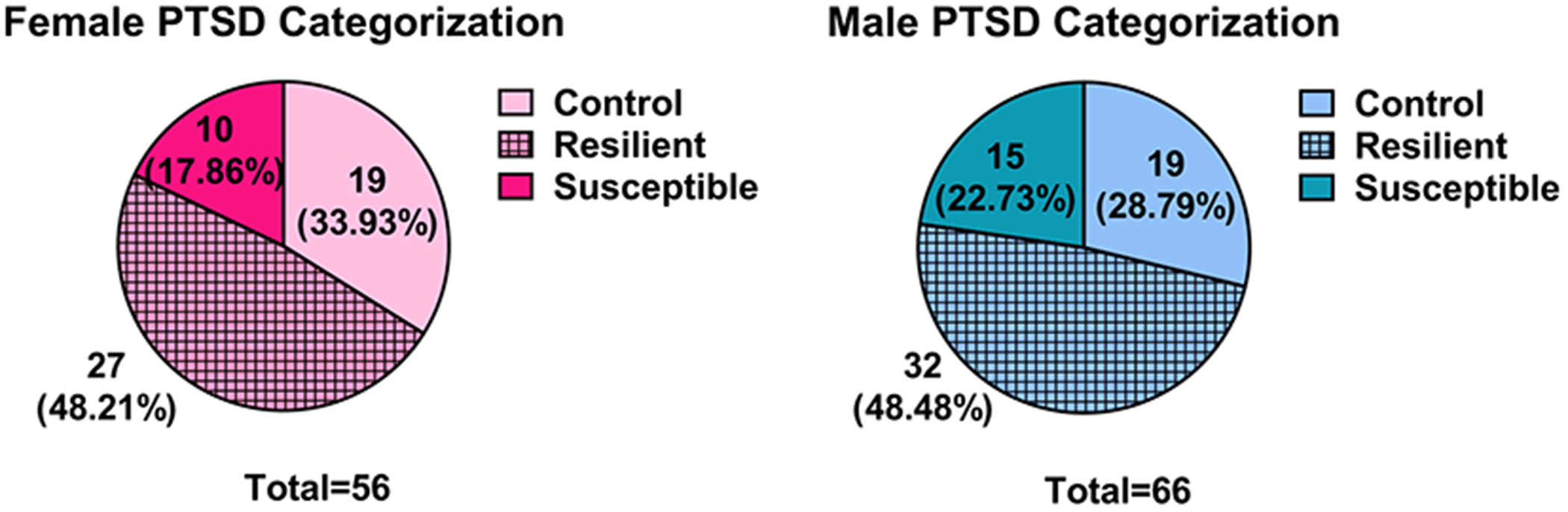
PTSD-Like Behavioral Phenotyping. Among the mice that underwent mSPS, 29.76% were determined to be susceptible. When segregated based on sex, 27.03% of female mSPS mice and 31.91% of male mSPS mice were classified as being susceptible.

After selecting the parameters to define the susceptible phenotype, absolute *z*-scores were calculated for each mouse for each behavioral parameter using the control mean and standard deviation (Andrade, 2021). A cutoff *z*-score of 1.44, corresponding to the 85% confidence interval, was utilized to identify mice with extreme behavior for each behavioral parameter. Mice who had an absolute *z*-score above 1.44 on at least one of the parameters for the DSM-V criteria of PTSD received one point for that criterion. Mice that demonstrated 5 points, one for each of the measurable DSM-V criteria of PTSD, were considered to have a susceptible phenotype (Table 1). mSPS mice with 4 or less points were defined as resilient. Absolute *z*-scores were also calculated for the control mice for each behavioral parameter, and none of the control mice received 5 points; therefore, none of the control mice were classified as susceptible. The other 3 criteria for PTSD according to the DSM-V (A, F, and H) were not part of the point system, as all the mSPS mice met those criteria based on the experimental design (criteria A: mice underwent the mSPS paradigm, F: behavioral testing was conducted 4 weeks post-mSPS, and H: the behavioral phenotype could not be attributed to substance misuse or another medical condition). Among the mice that underwent mSPS, 29.76% developed a susceptible phenotype (Figure 3). The sex-specific incidence of the susceptible phenotype was 27.03% in females and 31.91% in males.

### Evaluation of anhedonia and hyperlocomotion in resilient mice

**Figure 4.**
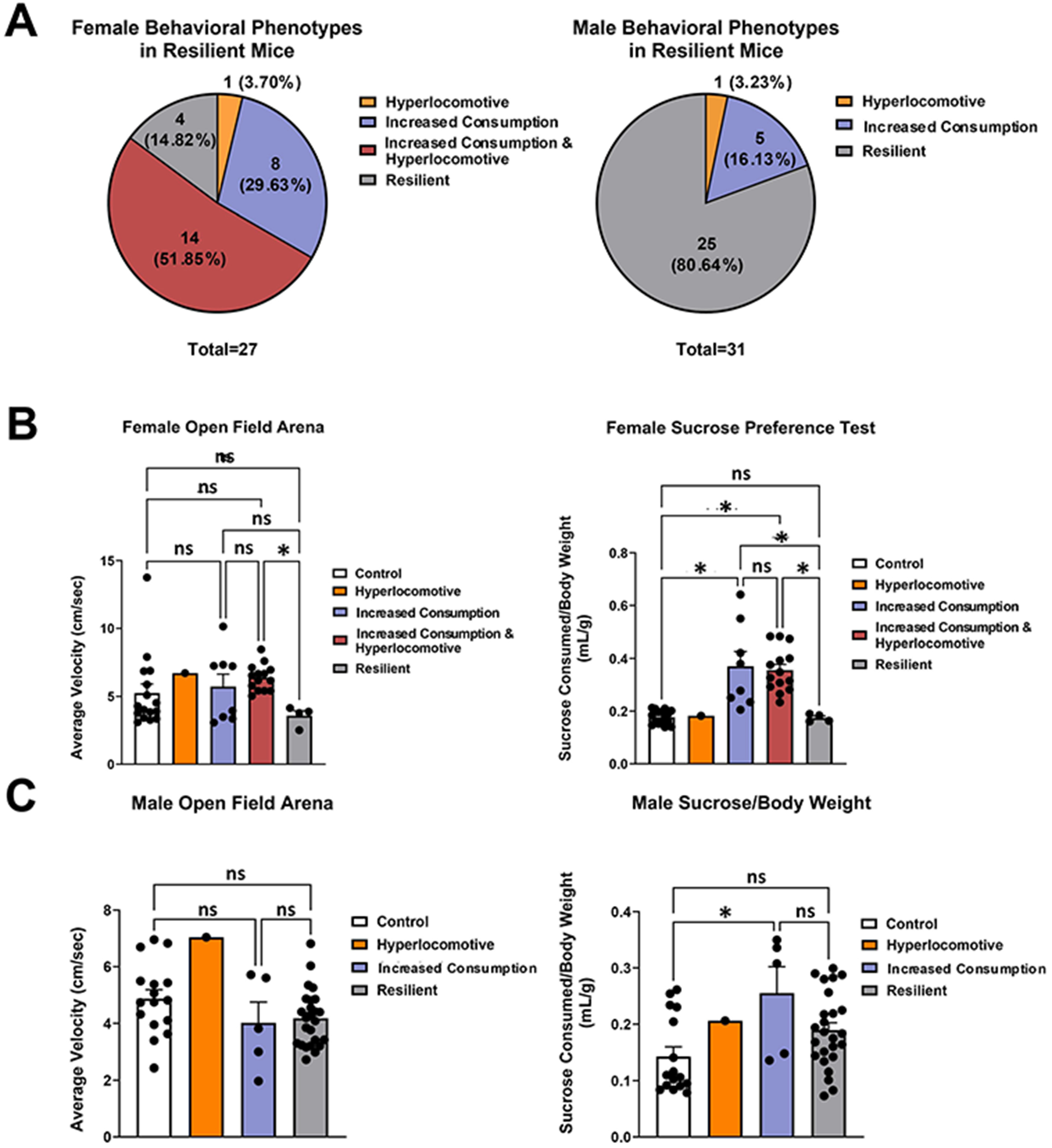
Evaluation of anhedonia and hyperlocomotion in resilient mice. Resilient C57BL/6NHsd were evaluated for anhedonic and hyperlocomotive phenotypes. The amount of sucrose consumed per body weight from the sucrose preference test was utilized to define anhedonic behavior. The average velocity in the open field arena was used to determine hyperlocomotive behavior. None of the resilient mice demonstrated decreased sucrose consumption associated with anhedonia but instead showed increased aberrant sucrose consumption. (A) Among the resilient mice, 22.41% displayed increased consumption behavior, 3.45% exhibited hyperlocomotive behavior, 24.14% had both increased consumption and hyperlocomotive behavior, and 50% had neither increased consumption nor hyperlocomotion. When segregating resilient mice based on sex, 29.63% of the female mice had increased consumption behaviors, 51.85% had increased consumption and hyperlocomotive behaviors, one mouse (3.7%) had hyperlocomotion, and a few of the mice displayed neither increased consumption nor hyperlocomotion (14.82%). Whereas most of the resilient male mice displayed neither increased consumption nor hyperlocomotion (80.64%), a few mice exhibited increased consumption behaviors (16.13%), and one mouse displayed hyperlocomotion (3.23%). (B) Female mice that had both increased consumption and hyperlocomotive phenotypes were hyperlocomotive in the open field arena when compared to resilient female mice. Whereas female resilient mice with increased consumption and both increased consumption and hyperlocomotive phenotypes consumed more sucrose solution per body weight as compared to control animals (data are displayed as the mean ± SEM, one-way ANOVA, *p value < 0.05, control females *n* = 16, increased consumption females *n* = 8, increased consumption and hyperlocomotive females *n* = 14, hyperlocomotive females *n* = 1 (removed from statistical testing), resilient females *n* = 4). (C) There was no significant difference in the average velocity of resilient male mice in the open field arena. Whereas, resilient male mice with increased consumption phenotype consumed more sucrose per body weight in the sucrose preference test than control mice (data displayed as mean ± SEM, one-way ANOVA, *p-value < 0.05, control males *n* = 16, hyperarousal males *n* = 1 (removed from statistical testing), increased consumption males *n* = 5, resilient males *n* = 25).

Many of the mice that were resilient to mSPS, as determined by behavioral testing at 4 weeks post-mSPS, displayed behavioral disruptions in some behavioral parameters. Further investigations into their behavior were conducted to assess anhedonia and hyperlocomotion. Interestingly, none of the resilient mice displayed canonical anhedonic behavior of decreased sucrose consumption. Instead, a subset of the resilient mice exhibited an increase in sucrose consumption and were classified as an increased consumption phenotype. Thus, of the resilient mice, 22.41% displayed increased consumption, and 3.45% displayed hyperlocomotive behavior (Figure 4A). A subset of the resilient mice, 24.14%, displayed both increased consumption and hyperlocomotive behaviors, and half of the resilient mice, 50%, exhibited neither increased consumption nor hyperlocomotive behavior (Figure 4A). Interestingly, when the sexes were separated, resilient female mice had the largest variety of different behavioral phenotypes where most of these mice displayed increased consumption and hyperlocomotion (51.85%), some showed increased consumption behavior alone (29.63%), one mouse demonstrated hyperlocomotion alone (3.7%), and a few mice had neither increased consumption nor hyperlocomotive phenotypes (14.82%) (Figure 4A). Whereas, most resilient male mice had neither increased consumption nor hyperlocomotive phenotypes (80.64%), with a few mice demonstrating increased consumption behaviors (16.13%) and one mouse showing hyperlocomotion (3.23%) (Figure 4A). Female mice with increased consumption and hyperlocomotive behaviors displayed increased average velocity when compared to the resilient mice in the open field arena, which corresponded with their behavioral phenotype categorization (Figure 4B). Male and Female mice categorized as having both ‘increased consumption’ and ‘increased consumption and hyperlocomotive’, consumed more sucrose per body weight (Figure 4B,C). Male mice did not show a significant increase in mean velocity in the open field arena (Figure 4C).

## Discussion

A major barrier in the field of PTSD basic science research is the lack of a standardized analysis method for defining a susceptible phenotype in traumatized mice (Richter-Levin et al., 2019; Verbitsky et al., 2020). This study aimed to provide a comprehensive analysis model of PTSD-like behavior that recapitulated the human phenotype using all diagnostic criteria for PTSD in the DSM-V, weighting each criterion evenly in the classification of the susceptible phenotype while also incorporating the RDoC constructs. Of the 8 criteria of PTSD in the DSM-V, 5 were measured through behavioral testing (Table 1, criteria B, C, D, E, and G), whereas the other 3 criteria (Table 1, criteria A, F, and H) were addressed through the experimental design. The 5 criteria assessed through behavioral testing also address the 4 RDoC constructs altered in PTSD (Table 2). The analysis methodology described in this study provides a simple and effective way to separate resilient and susceptible phenotypes in mSPS mice. The incidence of the susceptible phenotype in our mSPS mice was 29.76% (Figure 3), which is proportionally similar to that seen in the human population, where approximately 25% of individuals who experience a traumatic event develop PTSD in their lifetime (Grinage, 2003; Ressler et al., 2022).

There are a variety of models used to induce as well as define PTSD-like phenotypes in the field, leading to significant variability in the findings and conclusions (Deslauriers et al., 2018; Ferland-Beckham et al., 2021; Verbitsky et al., 2020). Social stressor models consist of housing instability, early-life stress, and social defeat and represent traumatic social experiences leading to PTSD-like symptoms. However, these models can be difficult to execute consistently because of the variability in rodent aggression and female aggressive behaviors (Borghans & Homberg, 2015; Verbitsky et al., 2020). Psychological stress paradigms consist of predator-based stress and involve exposing rodents to predator scents that rely on natural instincts to induce traumatization. These models frequently separate resilient from susceptible animals; however, reproducibility can be challenging due to variations in predator scents used, length of exposure, and lack of extreme behavioral responses in female rodents (Sarapultsev et al., 2024; Verbitsky et al., 2020). Physical stressor models include inescapable foot shock (IFS), underwater trauma, and prolonged restraint. These models are simple to execute and scale and translate to near death experiences often seen in soldiers (Borghans & Homberg, 2015; Sidles et al., 2024; Verbitsky et al., 2020). In the present study, we utilized an mSPS model that consisted of multimodal, single-day stress exposure incorporating aspects of physical, psychological, and physiological stress, comprising restraint stress, group forced swimming, and anesthesia-induced loss of consciousness followed by a chronic stress paradigm of single housing. Importantly, we combined the SPS model with repeated foot shock, adding a conditioned fear response on top of the sensitized stress response (Liberzon et al., 1997; Sidles et al., 2024; Wang et al., 2008; Wang et al., 2012; Xi et al., 2023).

The National Institute of Mental Health (NIMH) has attempted to address these variabilities in neuropsychiatric research by developing an RDoC framework that integrates neurobiology, neurocircuitry, and observable behavior to address each of the 5 constructs that form the foundation of neuropsychiatric disorders (Insel et al., 2010; Morris & Cuthbert, 2012). It has been identified in PTSD patients that 4 out of the 5 RDoC constructs are altered, which include negative valence, positive valence, cognitive processes, and arousal (Richter-Levin et al., 2019; Schmidt & Vermetten, 2018). Thus, it is encouraged that researchers studying PTSD preclinically utilize these 4 constructs when developing and analyzing PTSD-like animal models.

In addition to the variability in experimental design, the assessment and quantification of behavioral analysis are highly variable. The novel analysis employed in this study utilizes effect size in order to identify behavioral parameters that separated susceptible and resilient mice. The effect size quantifies the magnitude of difference between two groups. Thus, in reference to this study, the effect size highlighted the behavioral parameters that detected shifts in behavior between the mSPS group and the control group. Although not frequently used, effect size is gaining popularity in the statistical analysis of biological data, as it is capable of identifying biologically significant relationships as opposed to *p*-values, which are heavily influenced by statistical power (Maher et al., 2013; Moulin et al., 2016).

In order to identify individual mice with susceptible phenotypes, *z*-scores were then generated for each individual mSPS mouse for each behavioral parameter. This allowed the identification of specific mSPS mice with abnormal behavior in each behavioral test (Saulière et al., 2019). Composite *z*-scores have been used to define complex behavioral phenotypes in mice based on data from multiple behavioral parameters (Guilloux et al., 2011; Kraeuter, 2023; Labots et al., 2018). To generate a composite *z*-score, the *z*-scores from each individual behavioral parameter were averaged together (Song et al., 2013). Since the magnitude of individual *z*-scores can vary based on how abnormal a mouse’s behavior is to a specific behavior parameter (how many standard deviations their behavior is from the mean), this can lead to bias in the composite *z*-score in which certain behavioral parameters are incidentally weighted more heavily. Thus, in this study, a cutoff of the 85th confidence interval (*z*-score = 1.44) was utilized to identify mice with extreme behavior in each behavioral test. Mice must be above the 85th confidence interval for each of the 5 measurable DSM-V criteria in order to be classified as susceptible. This allows each criterion to be weighted equally and removes the intrinsic bias that is generated when a composite *z*-score is utilized. Moreover, this more closely mimicked the clinical diagnosis of PTSD, in which the DSM-V criteria of PTSD are weighted evenly and patients must suffer from symptoms from each criterion in order to obtain a PTSD diagnosis (Blevins et al., 2015).

Furthermore, this study considered sex differences in mouse behavior, which are often overlooked yet have long been described in the PTSD epidemiological literature. Although it has been established that females have a higher incidence of PTSD following a traumatic experience, the characterization of sex differences in symptomology is still debated (Hanáková et al., 2024; Kilpatrick et al., 2013; King et al., 2013; Olff, 2017). Some researchers have described reports of increased distress to traumatic reminders, avoidance symptoms, and hyperarousal in women, whereas other studies have noted that men experience hypervigilance and emotional numbing (Birkeland et al., 2017; Fullerton et al., 2001; King et al., 2013; Stuber et al., 2006). Alternatively, Chung et al. described little or no difference in PTSD symptomology based on sex and discovered stronger correlations between PTSD symptomology and the type of trauma induced (Chung & Breslau, 2008). Our analysis method for mice that underwent the mSPS paradigm supported the clinical studies noting sex differences in PTSD symptomology, demonstrating differential responses in male and female mice to each behavioral assessment.

Behavioral changes in the mSPS and SPS models often conflict across studies, which could be explained by using different mouse strains, early versus protracted time points of behavioral assessment, or methodological differences in the SPS procedure itself. Interestingly, in the present study, mSPS mice demonstrated increased risk-taking behavior in the elevated plus maze. Although not an expected finding in the elevated plus maze, as some studies report decreased or no change in open arm time in SPS mice (Fulco et al., 2022; O’Hara et al., 2025; You et al., 2021), it has been reported that chronically stressed mice exhibit increased risky behavior (Friedman et al., 2017; Mudra Rakshasa & Tong, 2020). These findings mimic human behavior, in which PTSD patients are known to engage in risky, self-destructive behaviors such as substance use, self-harm, disordered eating, and risky sexual or aggressive behaviors (Weiss et al., 2015). While we did not observe overall changes in light/dark box behavior, as others have reported in the SPS model (Tanaka et al., 2018), the induction paradigm reduced sociability in our male mSPS mice, which is also consistent with other reports in the literature (Azevedo et al., 2020; Ben-Azu et al., 2025; O’Hara et al., 2025). Additionally, it was unexpected that the mSPS mice in this study consumed higher amounts of sucrose solution and higher amounts of water during the habituation phase of the sucrose preference test than the control mice. Generally, a decrease in sucrose consumption is considered a marker of depressive-like behavior and anhedonia. However, there have been reports in the literature of increased sucrose consumption and polydipsia following chronic stress (Aranđelović et al., 2024; Gharibeh et al., 2022; Goto et al., 2014; Gross & Pinhasov, 2016; Torres Muñoz & Franklin, 2022). These reports support clinical data in which binge eating/drinking high-sugar and high-fat foods have been associated with PTSD and depression (Hirth et al., 2011; Mason et al., 2014; Zhang et al., 2024). Moreover, polydipsia has also been reported in concurrent psychiatric illnesses (Boyd, 1990; Evenson et al., 1987; Siegel, 2008; de Leon et al., 1994). Thus, although these findings are not always consistent with previous studies using the SPS mouse model, they are consistent with clinical evidence for behavioral changes in risk-taking, arousal, and social disturbances in individuals with PTSD.

Finally, similar to human patients, failing to score in every DSM-V criterion prevents our mice from qualifying for a susceptible phenotype. However, this does not indicate that they fail to show significant behavioral effects of fear and stress. As such, the resilient mice in our study were further examined for other behavioral changes associated with trauma, such as anhedonia and/or hyperlocomotion. Depression and generalized anxiety are frequently observed in humans following a traumatic event, with more women than men experiencing depression and/or anxiety (Bell et al., 2018; Grand et al., 2023; Luxton et al., 2010; Shalev et al., 1998; Vogt and Mangan, 2025; Wiseman et al., 2015). Interestingly, none of the resilient mice in this model displayed the canonical anhedonic phenotype of decreased sucrose consumption. Instead, some of the resilient mice demonstrated an increase in sucrose consumption, which, although atypical, has been shown by other research groups (Aranđelović et al., 2024; Gharibeh et al., 2022; Goto et al., 2014; Gross & Pinhasov, 2016; Torres Muñoz & Franklin, 2022). Furthermore, resilient mice in this model display a sex-dependent bias toward increased sucrose consumption or hyperlocomotion in females. Although hyperlocomotion may be a symptom of anxiety, it does not define the entire phenotype alone. Resilient mice that exhibited hyperlocomotion did not have heightened avoidant behavior, which is a hallmark of an anxiety-like phenotype in mice (Sartori et al., 2011). It has been reported in the literature that hyperlocomotion can interfere with behavioral assessments that measure anxiety-like behavior, thus masking a potential anxiety-like phenotype (Strekalova et al., 2005). Thus, although 27.59% of resilient mice displayed hyperlocomotion, it is unclear whether these mice also exhibit anxiety-like behaviors.

## Limitations

While this model and novel analysis strategy define a robust PTSD-like phenotype in mice, it is not without limitations. This mouse model utilizes a single day of trauma followed by mild chronic stress. This does not recapitulate other types of trauma that may lead to PTSD in humans, such as sexual and childhood abuse (Borghans & Homberg, 2015; Verbitsky et al., 2020). Future studies applying this analysis method of defining susceptible behavior using mice that have undergone other types of traumatization will be necessary to understand the generalizability of the analysis method.

## Summary

Overall, the experimental and analytical design of this study using a mSPS mouse model, provides a means to identify a reproducible and robust susceptible phenotype in mice that meets all 8 criteria of PTSD defined in the DSM-V and encapsulates the constructs of the RDoC matrix. Together, this provides a simple yet effective way of inducing and defining resilient and susceptible phenotypes, which can be utilized by those in the neuroscience field, as well as others, to better understand the underlying mechanisms driving the behavioral and biological changes that occur with PTSD. Importantly, this model design may also provide future opportunities to examine how PTSD interacts with other disease comorbidities, allowing greater strides to be made in understanding and treating PTSD.

## Figures and Tables

**Table 1.** Behavioral assessments.

DSM-V criteria	Symptoms	Behavioral assessment		Parameters	Point score
A	Psychological exposure	Mice underwent the mSPS paradigm	Experimental design	–	–
B	Intrusive thoughts	Conditioned Stimulus reminder test	Behavioral test	Pre-conditioned stimulus duration inactive (%) Post-conditioned stimulus duration inactive (%)	1
C	Avoidance of stimuli	Elevated plus maze Light/dark box	Behavioral test	Ratio of duration in open vs. closed arms Duration in dark side (s)	1
D	Negative alterations in cognition	Barnes maze Sucrose preference test	Behavioral test	Completed vs. failed probe trial Water consumed in 48 h (mL) Sucrose consumed in 24 h (mL)	1
E	Alterations in arousal and reactivity	Elevated plus maze Light/dark box	Behavioral test	Ratio of duration in open vs. closed arms Duration in dark side (s)	1
F	Psychological effects last at least 30 days	Behavioral testing was conducted 4-weeks post-mSPS	Experimental design	–	–
G	Social impairment	Crawley’s sociability assay	Behavioral test	Duration with novel mouse trial 2 (s) Latency to approach novel mouse trial 3 (s)	1
H	Behavioral phenotype could not be attributed to substance misuse or a medical condition	Behavioral testing was utilized to assign the PTSD-like phenotype	Experimental design	–	–
**Sum of points**		**Resilient < 5**	**PTSD-Like = 5**		

Mice underwent behavioral testing 4-weeks after being exposed to modified single prolonged stress (mSPS) to assess the presence of a PTSD-like phenotype. Each behavioral test was uniquely chosen to measure each of the criteria of PTSD according to the DSM-V. Mice received one point for each criterion if their *z*-score was above the 85% confidence interval (*z*-score = 1.44). Mice with at least 5 points (one point from each criterion) were determined to have a PTSD-like phenotype. Those with fewer than 5 points were classified as resilient.

**Table 2.** National Institute of Mental Health (NIMH) Research Domain Criteria (RDoC) classifications.

RDoC construct	Description	Behavioral assessment
Negative valence	Aversive situations and fear responses	Conditioned stimulus reminder test Elevated plus maze Light/dark box
Positive valence	Positive motivational situations and contexts	Sucrose preference test
Cognitive processes	Processes involved in attention, perception, memory, and language	Barnes maze Crawley’s sociability assay
Arousal	Neural systems involved in homeostatic regulation	Elevated plus maze Light/Dark Box

We chose behavioral DSM-V-based assessments classified according to the NIMH RDoC constructs.

## Data Availability

Data in this manuscript will be made available upon reasonable request.
